# *Lysimachia christinae* Hance Extract Mitigates Kidney Stone Formation: Association with NOX2/ROS Axis Modulation and Ferroptosis

**DOI:** 10.3390/cimb48050520

**Published:** 2026-05-16

**Authors:** Lian Xia, Zhaoguo Zhou, Chen Luo, Yan Yang, Daike Zou, Hanyue Zhang, Kaizhi Hu, Xianqin Luo

**Affiliations:** 1 College of Traditional Chinese Medicine, Chongqing Medical University, Chongqing 400016, China; x2023111957@outlook.com (L.X.); 15502322300@163.com (Z.Z.); morningstarluo@outlook.com (C.L.); 15348205631@163.com (Y.Y.); z2893426530@163.com (D.Z.); 2 Chongqing Key Laboratory of Traditional Chinese Medicine for Prevention and Cure of Metabolic Diseases, Chongqing 400016, China; 3 Chongqing Institute of Medicinal Plant Cultivation, Chongqing 408400, China; zhy0611735@163.com; 4 Hu Kaizhi National Veteran TCM Pharmacist Legacy Studio, Chongqing Institute of Medicinal Plant Cultivation, Chongqing 408435, China; 5 College of Chinese Materia Medica, Chongqing University of Chinese Medicine, Chongqing 402760, China

**Keywords:** *Lysimachia christinae* Hance, kidney stone, oxidative stress, ferroptosis

## Abstract

Kidney stone disease is a common urinary system disorder with a continuously rising global incidence, posing a major public health challenge. As a classic traditional Chinese medicine for the treatment of kidney stones, *Lysimachia christinae* Hance (LCH) has not yet been fully elucidated in terms of its pharmacological mechanism. In this study, a rat model of calcium oxalate kidney stones and a calcium oxalate monohydrate (COM)-induced injury model of human renal tubular epithelial (HK-2) cells were established. Combined with transcriptomic analysis and experimental verification, the therapeutic effect and underlying molecular mechanism of LCH against kidney stones were systematically explored. Results demonstrated that LCH extract significantly reduced serum levels of blood urea nitrogen (BUN) and creatinine (Cr), as well as renal tissue levels of kidney injury molecule-1 (KIM-1) and cystatin-C (Cys-C) in rats with calcium oxalate crystal-induced renal injury, and diminished calcium oxalate crystal deposition and adhesion in rat renal tissues as well as HK-2 cells, thus exerting a robust renoprotective effect. Mechanistically, transcriptome sequencing indicated that the anti-nephrolithiasis effect of LCH was closely related to the inhibition of oxidative stress and ferroptosis. LCH extract reversed CaOx crystal-induced upregulation of NADPH oxidase 2 (NOX2) and downregulation of superoxide dismutase 2 (SOD2), reduced intracellular oxygen species (ROS) levels, downregulated the expression of transferrin receptor 1 (TFR1) and acyl-CoA synthetase long-chain family member 4 (ACSL4) while upregulating that of ferritin heavy chain 1 (FTH1), solute carrier family 7 member 11 (SLC7A11) and glutathione peroxidase 4 (GPX4), and diminished intracellular iron accumulation, thereby effectively ameliorating crystal-mediated renal injury. The present study demonstrates that the therapeutic effect of LCH on kidney stones is closely related to the regulation of the NOX2/ROS signaling axis and ferroptosis, providing novel theoretical evidence for its clinical application.

## 1. Introduction

Kidney stones are a prevalent disorder in urology characterized by high incidence and recurrence rates [1]. Its global prevalence is on a continuous upward trend, with a nationwide prevalence ranging from 1.61% to 20.45% in China [2]. Calcium oxalate stones constitute the most common subtype, accounting for approximately 70–80% of all urinary calculi [3]. The formation of calcium oxalate stones begins with the supersaturation of oxalate and calcium ions in urine, followed by crystal nucleation and growth, and ultimately their retention and aggregation on the surface of renal tubular epithelial cells [4]. During this process, damage to renal tubular epithelial cells induced by high concentrations of oxalate or calcium oxalate crystals is considered the key initial step that triggers the entire stone formation cascade [5].

Oxalate and calcium oxalate crystals can induce excessive reactive oxygen species (ROS) production in renal tubular epithelial cells. Among these processes, NADPH oxidase 2 (NOX2) serves as the pivotal enzyme mediating such oxidative injury, whereas superoxide dismutase 2 (SOD2), a mitochondrial antioxidant enzyme, constitutes the primary defensive barrier against oxidative stress [6]. Accordingly, the imbalance between pro-oxidant and antioxidant systems represented by NOX2 and SOD2 is presumed to govern the extent of renal tubular cell injury and ultimate cellular fate under hyperoxaluric conditions [7]. Recent investigations have demonstrated that ferroptosis, a form of regulated cell death driven by excessive lipid peroxidation, is closely correlated with oxidative injury and is characterized by glutathione depletion, impaired glutathione peroxidase 4 (GPX4) activity, and mitochondrial structural aberrations [8,9]. In a calcium oxalate crystal-induced renal injury model, modulating the SLC7A11/GPX4 signaling pathway can suppress crystal-associated ferroptosis [10]. Furthermore, the ferroptosis inhibitor Ferrostatin-1 can alleviate cellular damage and reduce stone formation [11]. These studies highlight the intricate relationship between ferroptosis and kidney stones.

Considering the central and synergistic roles of oxidative stress and ferroptosis in the progression of kidney stone-induced renal injury, it is of great clinical significance to identify effective therapeutic agents that target these two pathological pathways. Accumulating preclinical evidence has validated that extracts derived from a panel of classic anti-urolithic traditional Chinese medicines—exemplified by Pyrrosiae Herba, Lygodii Spora, and Desmodii Styracifolii Herba—exert therapeutic effects against calcium oxalate kidney stones by suppressing oxidative stress and ferroptosis. Notably, a subset of these studies has further delineated the precise regulatory targets through which these herbal extracts act within these pathological cascades [12,13,14]. As a classic traditional Chinese medicine widely applied for diuresis and calculus removal, *Lysimachia christinae* Hance (LCH) has long been used in the clinical prevention and treatment of urinary system calculi [15]. Modern pharmacological studies have well-characterized the main bioactive components of LCH, including flavonoids, phenolic acids, and polysaccharides, which are the material basis for its pharmacological activities. Accumulating in vitro and in vivo studies have confirmed that LCH and its active extracts exert multiple biological effects, including prominent antioxidant, anti-inflammatory, diuretic, and renal tubular epithelial cell-protective activities [16]. In studies focused on the anti-kidney stone activity of LCH, existing reports have preliminarily confirmed that LCH treatment reduces calcium oxalate crystal deposition in rat renal tissues, improves core renal function markers including blood urea nitrogen (BUN) and serum creatinine (Scr), and attenuates renal tissue injury by enhancing endogenous antioxidant enzyme activity and lowering the level of the lipid peroxidation product malondialdehyde (MDA) [17]. Mounting evidence has confirmed that flavonoid-rich extracts from anti-urolithiasis herbs can alleviate renal injury by blocking the NOX2/ROS signaling cascade and inhibiting ferroptosis, which further supports the potential of LCH to exert similar regulatory effects in kidney stone injury [18]. However, despite the well-documented clinical efficacy and preliminary pharmacological effects of LCH, critical research gaps remain unaddressed, especially when compared with other well-studied anti-urolithiasis herbal extracts. Therefore, the present study established a calcium oxalate crystal induced renal injury model. Combined with transcriptomic analysis and comprehensive experimental verification, this work aimed to explore whether LCH could inhibit ferroptosis and exert renoprotective effects by modulating NOX2/ROS mediated oxidative stress. The findings will provide novel experimental evidence and a mechanistic basis for the clinical application of LCH in the prevention and treatment of kidney stones and secondary renal damage, and further enrich the pharmacological mechanism of LCH in the intervention of metabolic renal diseases.

## 2. Materials and Methods

### 2.1. Materials

LCH was provided by the Chongqing Institute of Medicinal Plant Cultivation and authenticated by Professor Luo Xianqin of Chongqing Medical University. LCH was pulverized and passed through a 40-mesh sieve. The resulting powder was then soaked in distilled water (*w/v* = 1:8) for 1 h, followed by two consecutive decoctions (30 min each). The combined filtrates were concentrated to yield a crude extract using a rotary evaporator (Shanghai Yarong Biochemical Instrument Factory, Shanghai, China), subsequently lyophilized, and stored at 4 °C. Subsequently, ultra-performance liquid chromatography coupled with quadrupole time-of-flight mass spectrometry (UPLC–Q/TOF/MS, Agilent Technologies, Santa Clara, CA, USA) was employed for the chemical composition analysis of LCH extract. Ethylene glycol and ammonium chloride were purchased from Shanghai MacKlin Biochemical Technology Co., Ltd. (Shanghai, China). Assay kits for blood urea nitrogen (BUN), creatinine (Cr), kidney injury molecule-1 (KIM-1), cystatin-C (Cys-C), superoxide dismutase (SOD), malondialdehyde (MDA), hydrogen peroxide (H_2_O_2_), lactate dehydrogenase (LDH), and glutathione peroxidase (GSH-Px) were obtained from Elabscience Biotechnology Co., Ltd. (Wuhan, China). The BCA protein quantification kit was purchased from Beyotime Institute of Biotechnology (Shanghai, China). Osteopontin (OPN), cluster of differentiation 44 (CD44), NADPH oxidase 2 (NOX2), and superoxide dismutase 2 (SOD2) antibodies were obtained from CliniSciences Research Center Co., Ltd. (Nanjing, Jiangsu, China). Acyl-CoA synthetase long-chain family member 4 (ACSL4), Glutathione peroxidase 4 (GPX4), Transferrin receptor 1 (TFR1), Solute carrier family 7 member 11 (SLC7A11), and Ferritin heavy chain 1 (FTH1) antibodies were purchased from Huaan Biotechnology Co., Ltd. (Jinan, Shandong, China).

### 2.2. Animals and Treatment

To ensure a higher success rate of calcium oxalate kidney stone model establishment and a more stable stone formation phenotype, and to avoid interference from the estrogen-mediated anti-urolithic effect in female rats, 7-week-old male Sprague–Dawley (SD) rats weighing 200–240 g were purchased from the Experimental Animal Center of Chongqing Medical University. The animal production license number was SCXK (Yu) 2018-0003. The animals were housed under barrier-system conditions at the Experimental Animal Center of Chongqing Medical University with strictly controlled environmental parameters. The ambient temperature was maintained at 20–25 °C, relative humidity at 45–55%, and a 12 h light/12 h dark photoperiod cycle was applied. After one week of adaptive feeding, the rats were randomly divided into five groups (*n* = 6 per group, sample size compliant with animal welfare 3R principle and field norms), namely the control group, model group, low-dose LCH group (LCH-L, 1.5 g·kg^−1^), medium-dose LCH group (LCH-M, 4.5 g·kg^−1^), and high-dose LCH group (LCH-H, 13.5 g·kg^−1^). The LCH dose gradient was set based on clinical equivalent dose conversion in accordance with the Chinese Pharmacopoeia, with a 3-fold equal-ratio gradient to explore its dose-dependent renoprotective effect, and no toxic reactions were observed during the experiment. The whole experiment was divided into two sequential, non-overlapping phases: a 14-day calcium oxalate kidney stone model induction phase, followed by a 14-day therapeutic intervention phase. During the model induction phase, all rats except those in the control group received daily gavage of distilled water containing 2% (*v*/*v*) ethylene glycol and 1% (*w*/*v*) ammonium chloride for 14 consecutive days to establish the calcium oxalate kidney stone model. Rats in the control group were given standard laboratory chow and ad libitum access to normal drinking water throughout the entire experimental cycle. After successful model induction, the therapeutic intervention phase was initiated: the low-, medium-, and high-dose LCH groups were given the corresponding doses of LCH extract (1.5, 4.5, and 13.5 g·kg^−1^ body weight) by oral gavage once daily for 14 consecutive days, while the control and model groups were administered an equal volume of isotonic saline via the same route daily during this phase. At the end of the intervention phase, all rats were fasted with free access to water for 12 h after the final administration, then anesthetized for sample collection. Blood samples were harvested via the abdominal aorta, and renal tissue specimens were collected for subsequent analyses.

### 2.3. Biochemical Indicators

Serum concentrations of BUN and Cr, as well as renal tissue homogenate levels of KIM-1, Cys-C, LDH, MDA, H_2_O_2_, GSH-Px and SOD, were determined using commercial assay kits provided by Elabscience Biotechnology Co., Ltd. (Wuhan, China) in accordance with the manufacturer’s protocols.

### 2.4. RNA-Sequencing Analysis

Rat renal tissues preserved in liquid nitrogen were retrieved, and total RNA was extracted using TRIzol reagent (Beyotime Biotechnology, Shanghai, China). RNA integrity and purity were assessed via agarose gel electrophoresis and the Agilent 2100 Bioanalyzer (Agilent Technologies, Santa Clara, CA, USA). Samples with an RNA integrity number (RIN) ≥ 7.0 were selected for subsequent library construction. Transcriptome libraries were constructed with the NEBNext^®^ Ultra™ RNA Library Prep Kit for Illumina^®^ (New England Biolabs, Ipswich, MA, USA), with 1 μg of total RNA input per sample. Following purification, cDNA fragments of 250–300 bp in length were screened, amplified and enriched by PCR, followed by purification and quality control of the final libraries. High-throughput RNA sequencing was performed on the Illumina NovaSeq 6000 platform by Majorbio Bio-pharm Technology Co., Ltd. (Shanghai, China), and approximately 60 million clean reads (6 Gb data) were generated for each sample. After normalization of gene expression data, differentially expressed genes (DEGs) were screened using the edgeR package (version 3.40.2) with the threshold criteria of [Log2 (fold change)] ≥ 1.0 and false discovery rate (FDR) < 0.05. Functional enrichment analyses of DEGs were conducted based on Gene Ontology (GO) and Kyoto Encyclopedia of Genes and Genomes (KEGG) databases. Hierarchical clustering analysis was further performed to evaluate the similarity of gene expression profiles among samples.

### 2.5. Cell Culture and Treatment

Human renal tubular epithelial cell line HK-2 was obtained from Procell Life Science & Technology Co., Ltd. (Wuhan, China) and maintained in Dulbecco’s Modified Eagle Medium/Nutrient Mixture F 12 (DMEM/F12) supplemented with 10% fetal bovine serum at 37 °C under a 5% CO_2_ humidified atmosphere. To establish an HK-2 cell injury model, cells were pretreated with 200 μg·mL^−1^ calcium oxalate monohydrate (COM) [19] crystals for 2 h, followed by incubation with gradient concentrations of LCH extract (0, 2, 4, 8, 16, 32, 64, 128 μg·mL^−1^) for 24 h. All in vitro experiments were independently repeated three times to ensure the stability and reproducibility of the results.

### 2.6. Cell Viability Assay

Cell viability was determined using the Cell Counting Kit-8 (CCK-8) assay (Beyotime, Shanghai, China). HK-2 cells were seeded into 96 well plates and cultured until reaching 80% confluence. Following treatment with gradient concentrations of LCH extract for 2 h, cells were exposed to 200 μg·mL^−1^ COM for 24 h. The supernatant was discarded, and cells were rinsed twice with phosphate-buffered saline (PBS). Subsequently, 110 μL of CCK-8 working solution, consisting of 10 μL CCK-8 reagent and 100 μL basal medium, was added to each well. After incubation at 37 °C for 1 h, the absorbance was measured at 450 nm.

The levels of SOD activity and MDA content in HK-2 cells were determined using commercial assay kits obtained from Elabscience Biotechnology Co., Ltd. (Wuhan, China).

### 2.7. Measurement of Intracellular ROS and Lipid Peroxidation Level

HK-2 cells were initially exposed to 200 μg·mL^−1^ COM, followed by subsequent treatment with LCH extract at concentrations of 8, 16 and 32 μg·mL^−1^ for 24 h. For intracellular ROS detection, the treated cells were incubated in serum free DMEM/F12 medium supplemented with DCFH-DA (Beyotime, Shanghai, China) at 37 °C for 30 min, gently washed three times with PBS, and the fluorescence intensity of ROS was observed under a laser scanning confocal microscope; for lipid peroxidation assay, the treated cells were stained with 2 μM BODIPY 581/591 C11 probe (Beyotime, Shanghai, China) and incubated at 37 °C for 30 min under light protected conditions, rinsed three times with PBS, stained with DAPI-containing antifade mounting medium (Southern Biotech, Guangzhou, China) at room temperature for 5 min, and subsequently imaged by laser scanning confocal microscopy.

### 2.8. Histology and Immunohistochemical Staining

Renal tissues from different groups were fixed with 10% glutaraldehyde, embedded in paraffin, and cut into 5 μm-thick sections. Hematoxylin–eosin (HE), Pizzolato, and periodic acid-Schiff (PAS) staining were conducted to assess renal crystal deposition and renal tubular injury. For immunohistochemical staining, sections were incubated overnight at 4 °C with primary antibodies against CD44 (1:100), OPN (1:100), SOD2 (1:100) and NOX2 (1:200), followed by incubation with secondary antibodies (Beyotime, Shanghai, China). Subsequently, antibody staining was visualized using 3,3′-diaminobenzidine (DAB, Beyotime, Shanghai, China).

### 2.9. Measurement of ROS Level

Renal tissue ROS levels were determined using the dihydroethidium (DHE) assay kit (Elabscience Biotechnology Co., Ltd., Shanghai, China), and fluorescence imaging was performed under an Olympus IX83 fluorescence microscope (Olympus, Tokyo, Japan).

### 2.10. Western Blotting Analysis

Total proteins were extracted from rat renal tissues and HK-2 cells, and protein concentrations were quantified using the BCA assay. The lysed proteins were separated by SDS-PAGE and then transferred onto a polyvinylidene fluoride (PVDF) membrane (Merck Millipore, Darmstadt, Germany). The membranes were blocked with 5% skim milk at room temperature for 1 h, and then incubated with primary antibodies overnight at 4 °C, followed by incubation with corresponding secondary antibodies. Enhanced chemiluminescence (ECL) detection reagent kits (Beyotime, Shanghai, China) were used to visualize immunoreactive bands, which were captured by the LAS 4000 imaging system. Protein expression levels were normalized to β-actin and GAPDH. The primary antibodies used in this study were as follows: OPN (1:1000), CD44 (1:1000), β actin (1:1000), SOD2 (1:1000), NOX2 (1:1000), ACSL4 (1:2000), GPX4 (1:2000), TFR1 (1:2000), SLC7A11 (1:2000), FTH1 (1:2000), GAPDH (1:3000).

### 2.11. Statistical Analysis

Statistical analyses of validation experiments were performed using GraphPad Prism 8.3.0 (GraphPad Software, San Diego, CA, USA), while computational and transcriptomic analyses were conducted in R (version 4.2.2). Comparisons between two groups were performed using an unpaired t-test, whereas multiple groups were evaluated by one-way ANOVA. A *p*-value < 0.05 was considered statistically significant. The presented data are expressed as mean ± standard deviation (SD).

## 3. Results

### 3.1. Chemical Composition in LCH Extract Through UPLC–Q/TOF/MS

UPLC–Q/TOF/MS analysis identified a total of 21 major chemical constituents in LCH extract, including phenolic acids, flavonoid glycosides, and other bioactive components (Supplementary Figure S1). The representative compounds included chlorogenic acid, quercitrin, rutin, kaempferol-3-O-rutinoside, kaempferol-3-O-β-robinobioside, quercetin-3-O-rutinosyl-(1→2)-O-rhamnoside, and mauritianin (Supplementary Table S1), which may contribute to its renoprotective effects against kidney stones.

### 3.2. Protective Effects of LCH Extract Against Calcium Oxalate Kidney Stones in Rats

**Figure 1:**
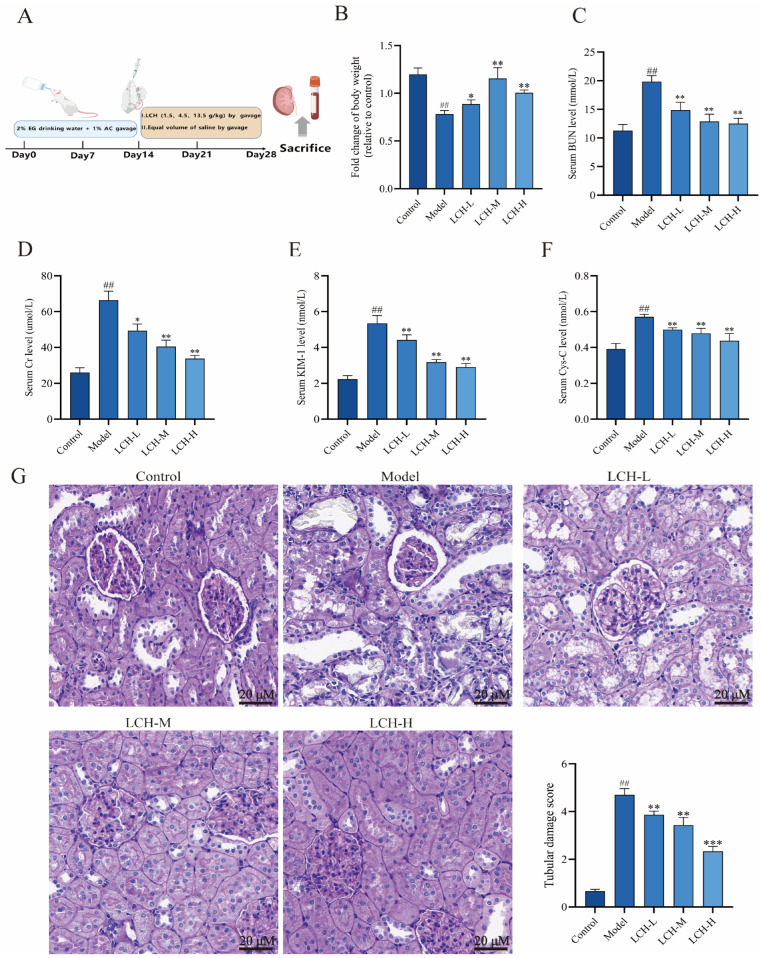
Protective effects of LCH extract against calcium oxalate kidney stones in rats. (**A**) Flow chart of animal experiments. (**B**) Change in weight. (**C**–**F**) Renal dysfunction was determined by levels of BUN, Cr, KIM-1 and Cys-C among the five groups. (**G**) PAS staining was used to illustrate and score tubular injury. ^##^ *p* < 0.01 compared to the Control. * *p* < 0.05, ** *p* < 0.01, *** *p* < 0.001 compared to the Model.

To evaluate the effects of LCH extract on rats with experimental kidney stones, a rat calcium oxalate kidney stone model was successfully established via combined induction with 2% ethylene glycol and 1% ammonium chloride. After 14 days of model induction, rats were intragastrically administered LCH at different doses (1.5, 4.5, and 13.5 g·kg^−1^) for therapeutic intervention (Figure 1A). The results revealed that LCH extract exerted a protective effect against body weight loss caused by calcium oxalate kidney stone formation in rats (Figure 1B). Renal function was assessed according to serum BUN and Cr levels. Compared with the control group, significantly increased BUN and Cr levels were observed in the model group, whereas treatment with LCH at varying doses alleviated renal function impairment (Figure 1C,D). Furthermore, the expression levels of renal tubular injury biomarkers KIM-1 and Cys-C in renal tissue were determined. Compared with the control group, model rats exhibited markedly upregulated KIM-1 and Cys-C expression, which was significantly downregulated following LCH treatment (Figure 1E,F). Renal tubular epithelial cells serve as the direct target of crystal toxicity. Once injured, these cells generate a pathological microenvironment that further facilitates heterogeneous nucleation and adhesion of crystals, thereby forming a vicious cycle. Accordingly, PAS staining was performed to evaluate the histological injury of renal tubules in rat renal tissue sections. Renal tubular injury was quantified via histological parameters including cellular loss, loss of brush border, and tubular lumen dilation. As illustrated in Figure 1G, LCH treatment markedly ameliorated renal tubular injury induced by calcium oxalate crystals. Collectively, these results demonstrate that LCH alleviates renal injury triggered by calcium oxalate crystal deposition.

### 3.3. LCH Extract Inhibits the Deposition and Adhesion of Crystals in Calcium Oxalate Kidney Stones in Rats

**Figure 2:**
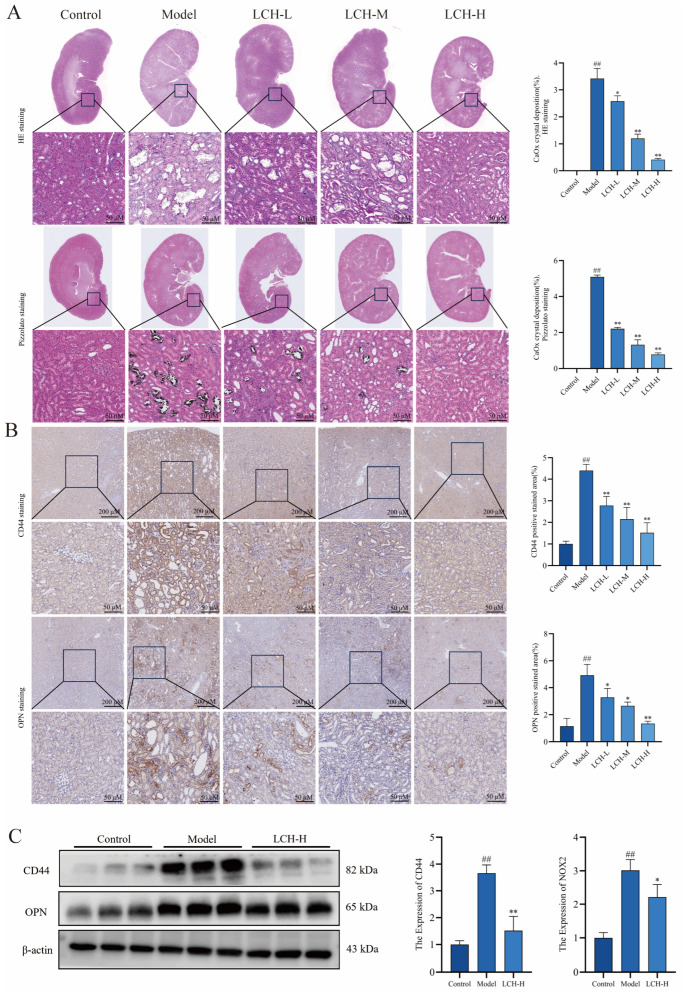
LCH extract inhibits the deposition and adhesion of crystals in calcium oxalate kidney stones in rats. (**A**) HE and Pizzolato staining followed by quantitative analysis. (**B**) IHC staining and quantitative analysis of CD44 and OPN. (**C**) CD44 and OPN expression were detected by Western blot. ^##^ *p* < 0.01 compared to the Control. * *p* < 0.05, ** *p* < 0.01 compared to the Model.

Observation of HE-stained sections revealed abundant calcium oxalate crystal deposition on renal tubular epithelial cells and inside tubular lumens in the model group relative to controls. Of note, all LCH intervention groups displayed markedly reduced crystal accumulation, among which the LCH-H group showed superior inhibition against calcium oxalate crystal formation compared with LCH-L group (Figure 2A). In addition, Pizzolato staining revealed that renal calcium oxalate crystals appeared as black punctate and aggregated deposits within renal tubular epithelial cells and tubular lumens. Relative to the model group, LCH treatment markedly alleviated renal calcium oxalate crystal deposition (Figure 2A). IHC analysis uncovered altered expression of adhesion molecules implicated in kidney stone pathogenesis. Specifically, CD44 and OPN levels were significantly elevated in model rats, highlighting their vital roles in crystal retention and kidney stone progression (Figure 2B). By contrast, LCH administration markedly downregulated CD44 and OPN expression. Consistently, Western blot assays further verified reduced protein abundance of OPN and CD44 upon LCH intervention (Figure 2C). Collectively, these findings demonstrate that LCH restrains the formation and deposition of renal calcium oxalate crystals in rats in a dose-dependent manner.

### 3.4. Effects of LCH Extract on Transcriptome in Calcium Oxalate Kidney Stone Rats

**Figure 3:**
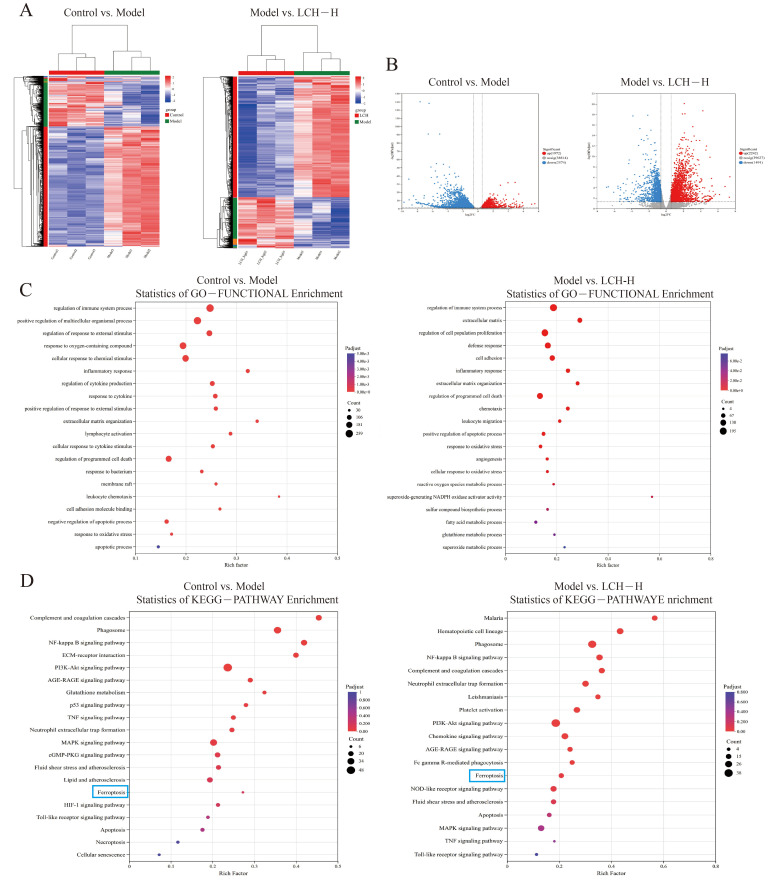
Effects of LCH Extract on Transcriptome in Calcium Oxalate Kidney Stone Rats. (**A**) Heatmap of DEG expression in kidney samples. (**B**) Volcano plots of DEG expression in kidney samples. (**C**) GO enrichment analysis bar plot. (**D**) KEGG pathway.

To investigate the therapeutic effects of LCH extract on kidney stones, transcriptome sequencing was performed in the control group, model group, and high-dose LCH group (*n* = 3 per group). The average mapping rate of clean reads to the reference genome was 97%, indicating high-quality sequencing data (Supplementary Table S2). Principal component analysis (PCA) showed clear separation between the three groups and good clustering of biological replicates within each group. Violin plots further demonstrated consistent overall distribution of gene expression levels across all samples (Supplementary Figure S2), collectively confirming the reliability of the transcriptome data. The heatmap revealed distinct separation of differentially expressed genes (DEGs) between the control and model groups, as well as between the model and LCH-H groups (Figure 3A). Volcano plots illustrated the differentially expressed genes between the control and model groups, as well as between the model and LCH-H groups (log2 fold change ≥ 1.0, adjusted *p*-value < 0.05, Figure 3B). A total of 4546 DEGs were identified between the control and model groups, among which 1972 were upregulated and 2574 were downregulated. In comparison, 3733 DEGs were detected between the model and LCH-H groups, with 2242 upregulated and 1491 downregulated. Among these DEGs, compared with the control group, the model group showed remarkable upregulation of the pro-oxidant gene NOX2, pro-ferroptotic gene ACSL4, and crystal adhesion molecule CD44. In contrast, ferroptosis suppressors GPX4, cystine transporter SLC7A11, and iron storage protein FTH1 were markedly downregulated.

To further explore the pathological mechanism of calcium oxalate kidney stones and the biological functions of differentially expressed genes modulated by LCH treatment, GO enrichment analysis was performed on 4546 DEGs between the control and model groups, and 3733 DEGs between the model and LCH-H groups. The results showed that DEGs between the control and model groups were mainly enriched in regulation of immune system process, response to oxygen-containing compound, inflammatory response, and cellular response to cytokine stimulus. In contrast, DEGs between the model and LCH-H groups were primarily enriched in regulation of programmed cell death, positive regulation of apoptotic process, response to oxidative stress, reactive oxygen species metabolic process, and glutathione metabolic process (Figure 3C). KEGG pathway enrichment and functional annotation analysis was performed to categorize the differentially expressed genes between the control and model groups, as well as between the model and LCH-H groups. The results revealed that DEGs between the control and model groups were significantly enriched in pathways including Phagosome, NF-κB signaling pathway, and Glutathione metabolism. In contrast, DEGs between the model and LCH-H groups were mainly enriched in Neutrophil extracellular trap formation, PI3K-Akt signaling pathway, and Chemokine signaling pathway. Notably, the ferroptosis pathway was markedly enriched following treatment with LCH extract (Figure 3D).

### 3.5. LCH Extract Attenuates Renal Oxidative Stress and Modulates NOX2/ROS Homeostasis

**Figure 4:**
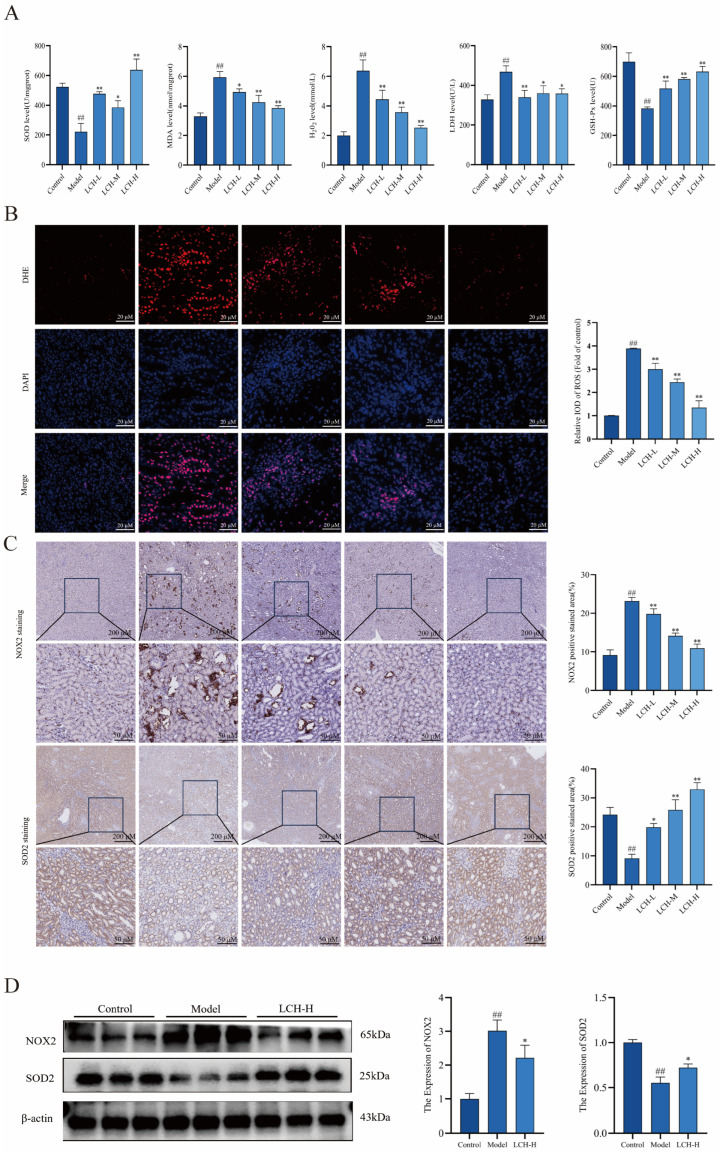
LCH extract attenuates renal oxidative stress and modulates NOX2/ROS homeostasis. (**A**) Expression levels of SOD, MDA, H_2_O_2_, LDH, and GSH-Px. (**B**) DHE staining to detect ROS expression levels in rats. (**C**) IHC staining and quantitative analysis of NOX2 and SOD2. (**D**) NOX2 and SOD2 expression were detected by Western blot. ^##^ *p* < 0.01 compared to the Control. * *p* < 0.05, ** *p* < 0.01 compared to the Model.

To investigate the regulatory effect of LCH extract on renal oxidative stress induced by calcium oxalate crystals, oxidative stress-related biochemical indices, ROS accumulation, and core regulatory protein levels in rat renal tissues were determined. Compared with the model group, LCH treatment markedly elevated the activities of SOD and GSH-Px, while reducing the levels of MDA, H_2_O_2_ and LDH activity (Figure 4A). These results indicated that LCH strengthened renal antioxidant defense and alleviated oxidative damage. DHE fluorescence assay revealed that the ROS fluorescence intensity in renal tissues was notably weakened in the LCH-treated group, demonstrating efficient scavenging of excessive ROS (Figure 4B). Furthermore, IHC staining and Western blot analysis verified that LCH extract significantly downregulated the protein expression of NOX2 and upregulated SOD2 (Figure 4C,D). Collectively, in calcium oxalate kidney stone rats, LCH inhibits NOX2-mediated excessive ROS production, restores SOD2-dependent antioxidant defense, maintains renal redox homeostasis, alleviates renal oxidative stress injury, and exerts protective effects against kidney stone formation.

### 3.6. LCH Extract Attenuates COM-Induced Injury in HK-2 Cells by Inhibiting Oxidative Stress

**Figure 5:**
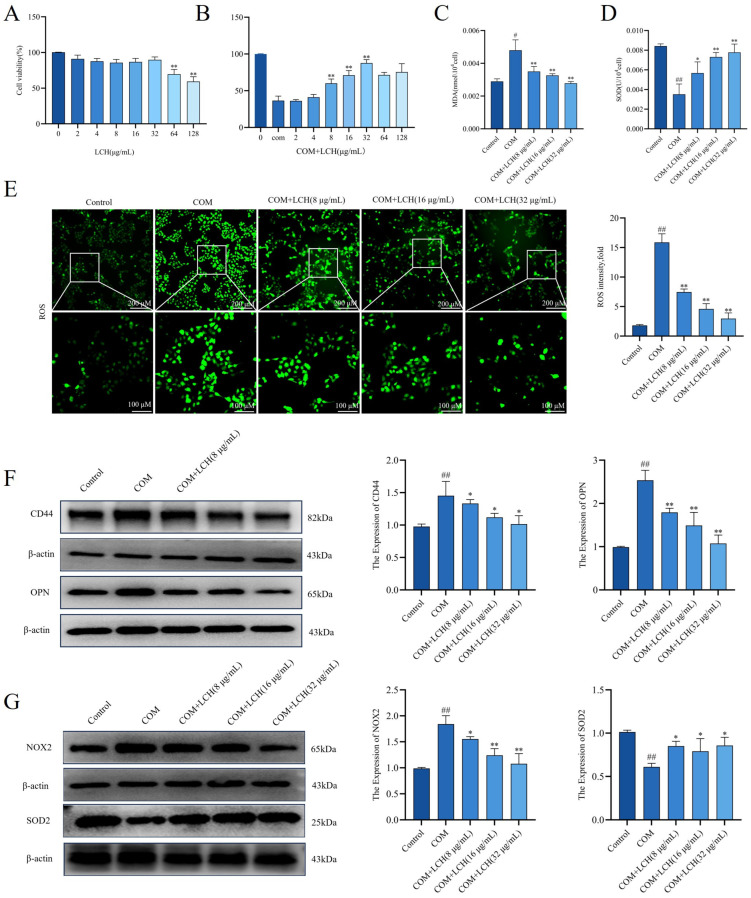
LCH extract attenuates COM-induced injury in HK-2 cells by inhibiting oxidative stress. (**A**) CCK-8 assay showing the effect of LCH extract (0–128 µg/mL) on HK-2 cells’ viability. (**B**) CCK-8 assay showing the restorative effect of LCH extract on COM-injured HK-2 cell viability. (**C**) MDA content. (**D**) SOD activity. (**E**) DCFH-DA staining to detect ROS production in HK-2 cells. (**F**) CD44 and OPN expression were detected by Western blot. (**G**) NOX2 and SOD2 expression were detected by Western blot. ^#^ *p* < 0.05, ^##^ *p* < 0.01 compared to the Control. * *p* < 0.05, ** *p* < 0.01 compared to the COM.

The effects of LCH extract on oxidative stress in COM-exposed HK-2 cells were explored. Initially, CCK-8 assay revealed that LCH extract at concentrations below 60 μg·mL^−1^ exhibited no obvious cytotoxicity to HK-2 cells (Figure 5A). In the COM-induced cell injury model, COM exposure markedly reduced cell viability, whereas LCH extract treatment at 8, 16 and 32 μg·mL^−1^ effectively reversed this decline (Figure 5B). Accordingly, these three concentrations were selected for subsequent cellular experiments. Detection of oxidative stress indices showed that LCH extract treatment significantly elevated SOD activity and decreased MDA levels (Figure 5C,D). DCFH-DA staining assay demonstrated that COM stimulation induced excessive intracellular ROS accumulation, which was notably suppressed by LCH extract intervention (Figure 5E). Furthermore, Western blot analysis indicated that LCH extract downregulated the expression of NOX2 and crystal adhesion molecules CD44 and OPN, while upregulating SOD2 expression in HK-2 cells (Figure 5F,G). Collectively, LCH extract alleviates COM-induced oxidative stress and cellular adhesion in HK-2 cells in a concentration-dependent manner.

### 3.7. LCH Extract Inhibits Calcium Oxalate Kidney Stone-Induced Ferroptosis

**Figure 6:**
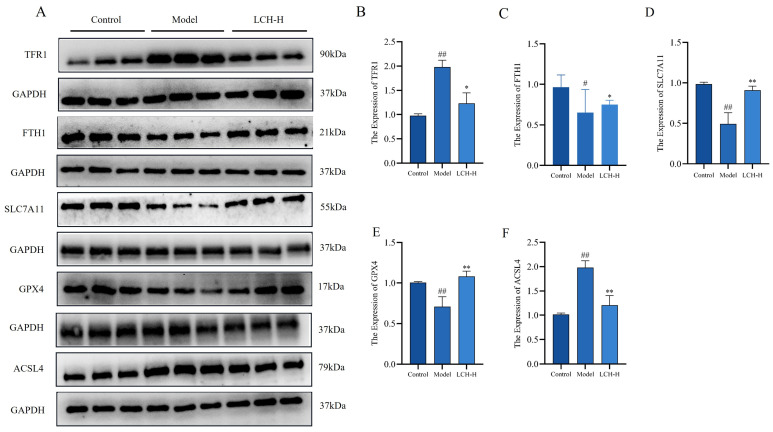
LCH extract inhibits calcium oxalate kidney stone-induced ferroptosis. (**A**) Protein expression of TFR1, FTH1, SLC7A11, GPX4 and ACSL4 in rat renal tissues was detected by Western blot. (**B**–**F**) Quantitative analysis of relative protein expression levels of TFR1 (**B**), FTH1 (**C**), SLC7A11 (**D**), GPX4 (**E**) and ACSL4 (**F**). ^#^ *p* < 0.05, ^##^ *p* < 0.01 compared to the Control; * *p* < 0.05, ** *p* < 0.01 compared to the Model.

To further validate the enrichment of the ferroptosis signaling pathway and the differential expression of candidate genes screened by transcriptome sequencing, Western blot analysis was performed to quantify the protein levels of core ferroptosis-related molecules (Figure 6). TFR1 and FTH1 serve as pivotal regulators of intracellular iron homeostasis. SLC7A11 functions as a critical subunit of the cystine–glutamate antiporter, and GPX4 acts as its downstream effector; together, these two proteins constitute the primary antioxidant defense system that mitigates lipid peroxidation. In addition, ACSL4 is a essential driver that facilitates the accumulation of lipid peroxides and accelerates ferroptosis progression. Western blot results demonstrated that, relative to the model group, the protein abundances of TFR1 and ACSL4 were markedly downregulated in the renal tissues of the LCH-H group. By contrast, the expression of FTH1, SLC7A11 and GPX4 was significantly upregulated in LCH-H treated rats. Collectively, these findings indicated that LCH intervention could restrain ferroptosis via modulating the NOX2/ROS signaling cascade, thereby exerting a protective therapeutic effect against kidney stone injury.

### 3.8. LCH Extract Alleviates COM-Induced Ferroptosis in HK-2 Cells by Regulating Intracellular Iron Homeostasis

**Figure 7:**
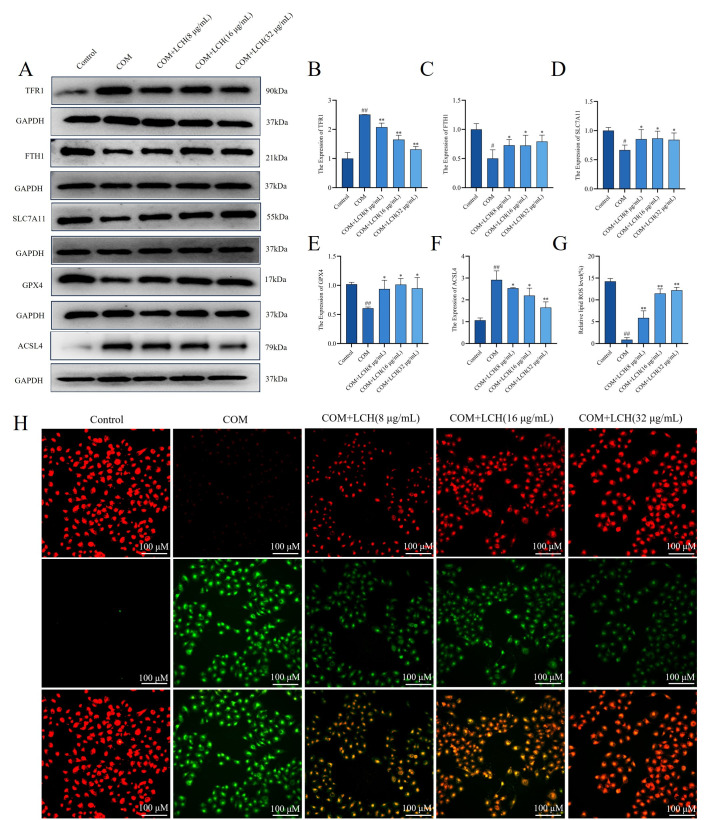
LCH extract alleviates COM-induced ferroptosis in HK-2 cells by regulating intracellular iron homeostasis. (**A**) Western blot detection of ferroptosis-related protein expression in COM-stimulated HK-2 cells. (**B**–**F**) Quantitative analysis of TFR1, FTH1, SLC7A11, GPX4 and ACSL4 protein levels. (**G**) Quantitative analysis of lipid peroxidation levels; (**H**) Lipid peroxidation staining results. ^#^ *p* < 0.05, ^##^ *p* < 0.01 compared to the Control. * *p* < 0.05, ** *p* < 0.01 compared to the Model.

Based on the above in vivo findings in rat renal tissues, we further validated the protective effect of LCH against COM-induced ferroptosis using an in vitro HK-2 cell injury model. Western blot results demonstrated that compared with the control group, the protein levels of the pro-ferroptosis markers TFR1 and ACSL4 were markedly upregulated in COM-stimulated HK-2 cells, while LCH intervention dose-dependently reversed this abnormal upregulation. In contrast, the protein expression of anti-ferroptosis and anti-lipid peroxidation regulators including FTH1, SLC7A11 and GPX4 was significantly downregulated in the COM model group, and LCH treatment effectively restored their expression levels (Figure 7A–F). Notably, cellular lipid peroxidation fluorescence staining was further performed to intuitively evaluate the antioxidant effect of LCH. The results showed that COM stimulation significantly increased cellular lipid peroxidation fluorescence intensity, whereas LCH pretreatment markedly suppressed COM-triggered lipid peroxidation accumulation in HK-2 cells (Figure 7G,H).

## 4. Discussion

Kidney stones represent a highly prevalent urological disorder with a substantial global disease burden. Southern China, one of the three major high-incidence regions worldwide, has witnessed a continuous increase in its prevalence over recent decades [20]. Although kidney stones are non-malignant, their complications, including urinary tract infections and urinary obstruction, can cause irreversible renal impairment and significantly compromise patients’ quality of life [21]. Traditional Chinese medicine (TCM) has a millennia-long history in the prevention and management of urolithiasis [22]. Among TCM herbs, LCH is one of the most widely prescribed agents for clinical stone expulsion [23]. Previous studies have demonstrated that flavonoid constituents of LCH inhibit excessive autophagy and apoptosis in renal tubular epithelial cells via the p38/MAPK signaling pathway, while its polysaccharide fractions exert diuretic effects to facilitate crystal excretion [24]. Nevertheless, the comprehensive molecular regulatory network underlying its anti-nephrolithic activity remains incompletely understood. This study reveals that the therapeutic effect of LCH against kidney stones is associated with the regulation of the NOX2/ROS axis and ferroptosis, providing a new mechanistic basis for the clinical treatment of kidney stones with LCH.

Calcium oxalate stones account for over 80% of all clinical kidney stones, and their pathogenesis is driven by a self-perpetuating vicious cycle of “crystal deposition—renal tubular epithelial injury—enhanced crystal adhesion” [25]. Renal tubular epithelial damage plays a decisive role in crystal–cell interactions, as injured cells exhibit disrupted membrane integrity and upregulated adhesion molecules, which in turn promote crystal adhesion and aggregation on the cell surface and accelerate stone formation [26,27]. In this study, rats with calcium oxalate nephrolithiasis showed significant elevations in serum renal function markers and urinary tubular injury markers, accompanied by extensive renal structural damage and widespread crystal deposition. Notably, LCH extract treatment effectively reversed these pathological changes, restored renal function, and attenuated histological injury. OPN, a major component of the kidney stone organic matrix, is frequently upregulated under pathological conditions. By binding to CD44 receptors aberrantly expressed on damaged renal tubular epithelial cells, OPN mediates crystal adhesion and retention on the tubular wall and contributes to the formation of Randall’s plaques, a critical initiating event in kidney stone development [28]. Consistent with these findings, we demonstrated that LCH treatment significantly downregulated CD44 and OPN expression both in vitro and in vivo, thereby reducing calcium oxalate crystal adhesion to renal tubular epithelial cells. Importantly, this effect is not only due to the direct inhibition of adhesion molecule expression but also secondary to LCH-mediated suppression of oxidative stress and ferroptosis, which collectively break the aforementioned vicious cycle of stone formation.

In calcium oxalate nephrolithiasis, accumulating clinical and preclinical evidence has established that ROS-mediated oxidative stress is a central driver of disease pathogenesis [29]. NOX2 is the predominant source of renal ROS production, and its aberrant activation drives excessive ROS accumulation, triggering lipid peroxidation and cellular damage [30]. The present study demonstrates that LCH significantly downregulates NOX2 expression while increasing SOD and GSH-Px activities and reducing MDA, H_2_O_2_ and LDH levels, thereby effectively restoring renal redox homeostasis and attenuating oxidative stress-induced renal injury. Notably, while previous studies only described LCH’s general antioxidant properties [31], our findings indicate NOX2 as a potential upstream mediator of its therapeutic effects, advancing our understanding of its anti-kidney stone mechanisms.

Intriguingly, our study demonstrates that the anti-nephrolithic effects of LCH are closely linked to ferroptosis inhibition and reduced intracellular lipid peroxidation. Ferroptosis, an iron-dependent form of programmed cell death, is a critical pathological mediator of nephrolithiasis-induced renal injury [32], and NOX2-mediated excessive ROS production directly triggers ferroptosis signaling pathway activation and accelerates renal tubular epithelial cell ferroptosis [33]. Accumulating evidence confirms that targeting ferroptosis represents a promising therapeutic strategy for calcium oxalate kidney stones; for instance, recent studies have shown that pharmacological activation of ALDH2 inhibits crystal-associated ferroptosis and protects renal tissue via the SLC7A11/GPX4 axis [34], however this intervention only targets the downstream antioxidant defense system of ferroptosis and fails to address upstream iron overload and lipid peroxidation initiation. Combining transcriptomic sequencing and molecular biology assays, we demonstrate that LCH blocks ferroptosis initiation by inhibiting TFR1 to reduce excessive iron uptake and downregulating ACSL4 to limit lipid peroxide production, while simultaneously upregulating FTH1 to enhance cellular iron storage capacity and alleviate intracellular free iron overload, and increasing SLC7A11 expression to restore cystine uptake and promote endogenous antioxidant synthesis. These coordinated effects collectively stabilize downstream GPX4 expression and function, efficiently clear excess lipid peroxides, and ultimately inhibit renal tubular epithelial cell ferroptosis.

Flavonoids constitute the core material basis for the anti-nephrolithic activity of LCH, among which quercetin, kaempferol, and luteolin are the most likely mediators of the observed biological effects. Quercetin has been confirmed to directly bind to and inhibit NOX2 activity, reduce ROS production, and suppress ferroptosis by modulating TFR1 and GPX4 expression [35]; kaempferol and luteolin exhibit potent antioxidant and anti-inflammatory activities, alleviating renal tubular epithelial cell injury and downregulating OPN expression [36,37]. However, functional validation of individual compounds was not performed in the present study, and the specific monomeric component or combinatorial effect of multiple constituents responsible for LCH’s therapeutic efficacy remains to be isolated and characterized. Furthermore, the core DEGs identified via RNA-seq were only validated at the protein level by Western blotting, lacking supplementary verification at the transcriptional level using qRT-PCR. Additionally, although we have demonstrated the regulatory effect of LCH on the NOX2/ROS pathway, direct causal evidence requires further strengthening due to the absence of NOX2-specific overexpression/knockout genetic intervention experiments and NOX2 inhibitor pharmacological control studies. These limitations should be addressed in future investigations to better define the material basis, safety profile, and clinical translational potential of LCH.

## 5. Conclusions

In conclusion, the renoprotective effect of LCH extract against calcium oxalate crystal-induced kidney stone injury is closely associated with modulation of the NOX2/ROS axis, suppression of oxidative stress, and regulation of ferroptosis in renal tubular epithelial cells. These findings offer novel theoretical insights and a scientific basis for the clinical application of LCH in the prevention and treatment of kidney stone diseases. Future work will focus on further verifying the molecular mechanism of LCH, and establishing long-term kidney stone models to better simulate clinical chronic progression and evaluate the long-term efficacy and recurrence-preventing potential of LCH.

## Data Availability

The original contributions presented in this study are included in the article/Supplementary Materials. Further inquiries can be directed to the corresponding author(s).

## Supplementary Materials

The following supporting information can be downloaded at: https://www.mdpi.com/article/10.3390/cimb48050520/s1.

## Abbreviations

The following abbreviations are used in this manuscript:

LCH	*Lysimachia christinae* Hance
COM	Calcium oxalate monohydrate
BUN	Blood urine nitrogen
Cr	Creatinine
Cys-C	Cystatin - C
KIM-1	Kidney Injury Molecule-1
HK-2	Human renal tubular epithelial cells
IHC	Immunohistochemistry
PAS	Periodic acid-Schiff staining
CD44	Cluster of differentiation 44
OPN	Osteopontin
HE	Hematoxylin-Eosin
CCK - 8	Cell counting kit-8
GO	Gene Ontology
KEGG	Kyoto Encyclopedia of Genes and Genomes
LDH	Lactate dehydrogenase
MDA	Malondialdehyde
SOD	Superoxide dismutase
GSH-Px	Glutathione peroxidase
PAGE	Polyacrylamidegel electrophoresis
PBS	Phosphate-buffered saline
PVDF	Polyvinylidene fluoride
ACSL4	Acyl-CoA synthetase long-chain family member 4
GPX4	Glutathione peroxidase 4
TFR1	Transferrin receptor 1
SLC7A11	Solute carrier family 7 member 11
FTH1	Ferritin heavy chain 1
