# Supplementary material for: Lysimachia christinae Hance Extract Mitigates Kidney Stone Formation: Association with NOX2/ROS Axis Modulation and Ferroptosis
Source: Curr Issues Mol Biol. 2026 May 16;48(5):520. doi: 10.3390/cimb48050520 (PMC13204593; doi:10.3390/cimb48050520)

Total ion current chromatogram: From top to bottom: positive mode, negative mode, and UV absorption spectrum at 254 nm.

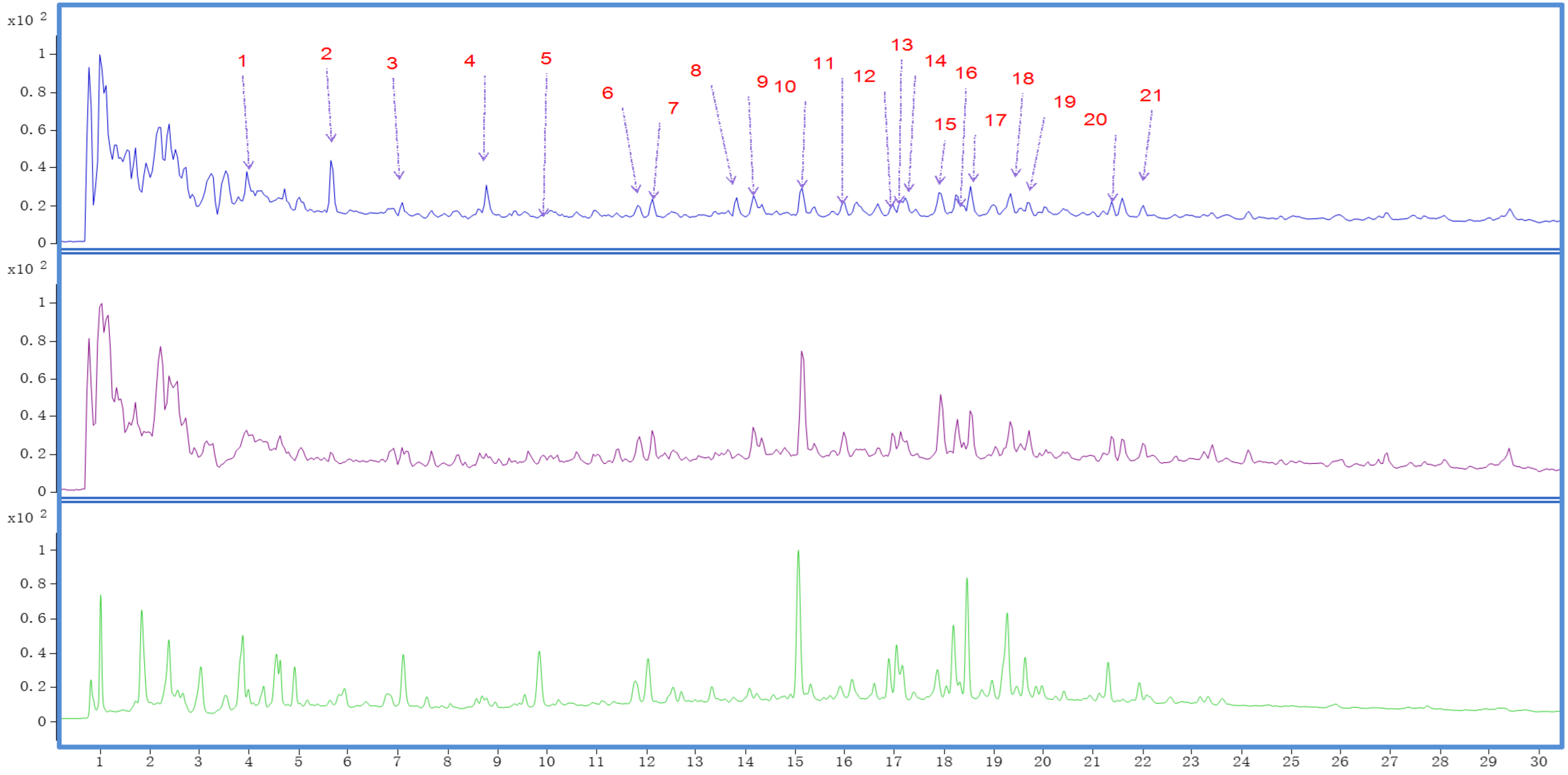

Supplement: Supplementary file 1 [file cimb-48-00520-s001.zip › Supplementary Figure S1.pdf]
