# Supplementary material for: Lysimachia christinae Hance Extract Mitigates Kidney Stone Formation: Association with NOX2/ROS Axis Modulation and Ferroptosis
Source: Curr Issues Mol Biol. 2026 May 16;48(5):520. doi: 10.3390/cimb48050520 (PMC13204593; doi:10.3390/cimb48050520)
Supplement: Supplementary file 1 [file cimb-48-00520-s001.zip › Supplementary Figure S2.pdf]

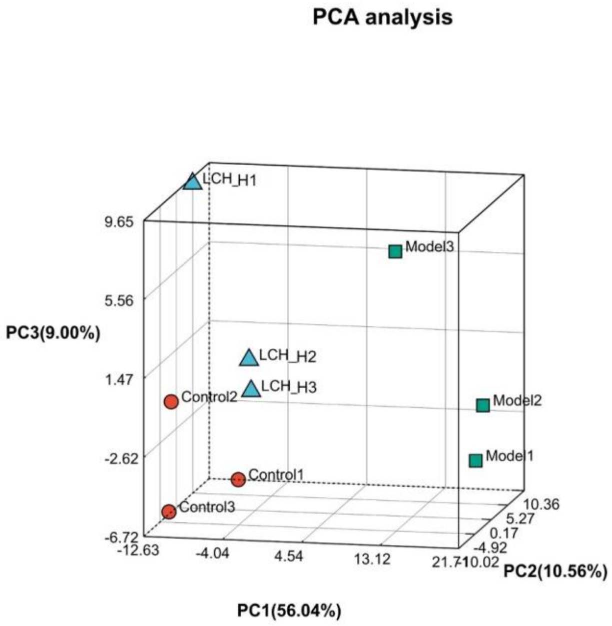

Results of PCA analysis

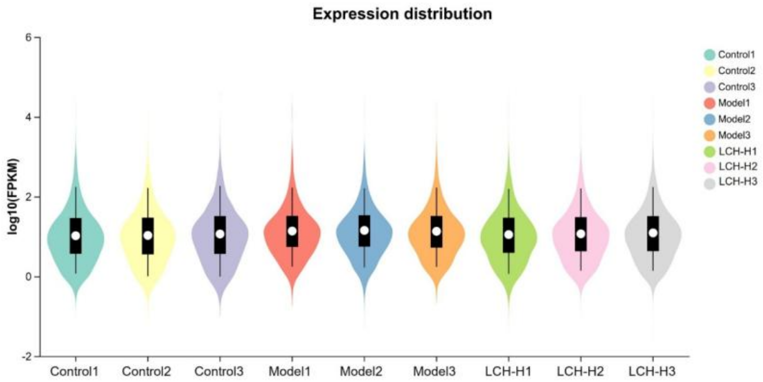

Violin plot of gene expression distribution
