# Supplementary material for: Lysimachia christinae Hance Extract Mitigates Kidney Stone Formation: Association with NOX2/ROS Axis Modulation and Ferroptosis
Source: Curr Issues Mol Biol. 2026 May 16;48(5):520. doi: 10.3390/cimb48050520 (PMC13204593; doi:10.3390/cimb48050520)
Supplement: Supplementary file 1 [file cimb-48-00520-s001.zip › Supplementary Table S1.pdf]

**Supplementary Table S1. The main compounds identified in LCH by UPLC-Q/TOF-MS.**

| NO | T(min) | $[M+H]^+$ | Error (ppm) | Formula | m/z | Compound name |
|---|---|---|---|---|---|---|
| 1 | 3.950 | $[M+H]^+$ | 0.76 | $C_8H_8O$ | 104.057 | P-methyl benzaldehyde |
| 2 | 5.645 | $[M+H]^+$ | 0.47 | $C_9H_{11}NO_2$ | 120.081 | L-Phenylalanine |
| 3 | 7.084 | $[M-H]^-$ | 0.01 | $C_9H_{17}NO_5$ | 146.082 | Pantothenic acid |
| 4 | 8.779 | $[M-H]^-$ | 1.0074 | $C_{11}H_{12}N_2O_2$ | 159.092 | L-Tryptophan |
| 5 | 9.881 | $[M+H]^+$ | 1.0095 | $C_{29}H_{26}O_3$ | 405.187 | Substituted polycyclic aromatic hydrocarbons |
| 6 | 11.830 | $[M+H]^+$ | 0.07 | $C_{16}H_{18}O_9$ | 191.056 | Chlorogenic acid |
| 7 | 12.127 | $[M+H]^+$ | 0.41 | $C_{22}H_{32}O_{15}$ | 213.075 | Forsythoside E |
| 8 | 13.823 | $[M+H]^+$ | 0.93 | $C_{17}H_{19}NO_3$ | 237.091 | Coclaurine |
| 9 | 14.163 | $[M+H]^+$ | 0.37 | $C_{19}H_{30}O_8$ | 385.187 | Roseoside |
| 10 | 15.139 | $[M-H]^-$ | 2.49 | $C_{21}H_{20}O_{11}$ | 359.078 | Quercitrin |
| 11 | 15.988 | $[M+H]^+$ | 0.33 | $C_{26}H_{28}O_{15}$ | 545.128 | Carlinoside |
| 12 | 16.960 | $[M+H]^+$ | -0.2 | $C_{33}H_{40}O_{20}$ | 611.160 | Clovin |
| 13 | 17.113 | $[M-H]^-$ | 0.37 | $C_{33}H_{40}O_{20}$ | 739.200 | Quercetin-3-O-rutinosyl-(1→2)-O-rhamnoside |
| 14 | 17.219 | $[M-H]^-$ | 1.09 | $C_{27}H_{30}O_{17}$ | 316.022 | Quercetin 3-gentiobioside |
| 15 | 17.898 | $[M+H]^+$ | 0.25 | $C_{26}H_{28}O_{14}$ | 433.092 | Vicenin -1 |
| 16 | 18.238 | $[M+H]^+$ | -0.61 | $C_{33}H_{40}O_{19}$ | 595.163 | Kaempferol 3-O- (2,6-α-L-dirhamnopyranosyl-β-D-glucopyranoside) |
| 17 | 18.535 | $[M-H]^-$ | -0.33 | $C_{33}H_{40}O_{19}$ | 575.142 | Mauritianin |
| 18 | 19.342 | $[M+H]^+$ | 0.56 | $C_{27}H_{30}O_{16}$ | 303.050 | Rutin |
| 19 | 19.681 | $[M+H]^+$ | 0.05 | $C_{27}H_{30}O_{15}$ | 287.055 | Kaempferol-3-O-rutinoside |
| 20 | 21.379 | $[M-H]^-$ | 1.55 | $C_{27}H_{30}O_{15}$ | 429.084 | Kaempferol-3-O-β-robinobioside |
| 21 | 22.015 | $[M-H]^-$ | 0.88 | $C_{15}H_{10}O_6$ | 284.032 | Cynaroside |
