# Supplementary material for: Lysimachia christinae Hance Extract Mitigates Kidney Stone Formation: Association with NOX2/ROS Axis Modulation and Ferroptosis
Source: Curr Issues Mol Biol. 2026 May 16;48(5):520. doi: 10.3390/cimb48050520 (PMC13204593; doi:10.3390/cimb48050520)
Supplement: Supplementary file 1 [file cimb-48-00520-s001.zip › Supplementary Table S2.pdf]

Alignment results of reads to the reference genome

Sample	Total reads	Reads mapped	Multiple mapped	Unique mapped
Control1	47526112	46264139(97.34%)	1644632(3.46%)	44619507(93.88%)
Control2	43820636	42666889(97.37%)	1503232(3.43%)	41163657(93.94%)
Control3	41730412	40879781(97.96%)	1416882(3.4%)	39462899(94.57%)
Model1	43427336	42150468(97.06%)	1342964(3.09%)	40807504(93.97%)
Model2	43908208	42791369(97.46%)	1278826(2.91%)	41512543(94.54%)
Model3	46398468	45200253(97.42%)	1422307(3.07%)	43777946(94.35%)
LCH_H1	41134120	40122414(97.54%)	1259382(3.06%)	38863032(94.48%)
LCH_H2	43965248	42927918(97.64%)	1534030(3.49%)	41393888(94.15%)
LCH_H3	46912718	45847127(97.73%)	1541395(3.29%)	44305732(94.44%)
